# Chinese Expert Consensus on Leptomeningeal Metastases of Lung Cancer

**DOI:** 10.1111/1759-7714.70088

**Published:** 2025-06-08

**Authors:** Gen Lin, Yongsheng Wang, Tao Xin, Ding Zhang, Qiuyu Zhang, Yangsi Li, Yudan Chi, Yun Fan, Anwen Liu, Haipeng Xu, Liang Shi, Longfeng Zhang, Qian Miao, Xiaobin Zheng, Lijun Li, Kaijia Zhou, Qiwei Yao, Zihua Zou, Kang Miao, Yaping Hong

**Affiliations:** ^1^ Department of Medical Oncology Beijing Tuberculosis and Thoracic Tumor Research Institute, Beijing Chest Hospital, Capital Medical University Beijing China; ^2^ Division of Thoracic Tumor Multimodality Treatment Cancer Center and National Medical Products Administration key Laboratory for Clinical Research and Evaluation of Innovative Drugs, West China Hospital, Sichuan University Chengdu Sichuan China; ^3^ Department of Oncology The Second Affiliated Hospital, Harbin Medical University Harbin China; ^4^ Department of Pulmonary and Critical Care Medicine Huashan Hospital, Fudan University Shanghai China; ^5^ Institute of Immunotherapy, School of Basic Medical Sciences, Fujian Medical University Fuzhou China; ^6^ Guangdong Lung Cancer Institute, Guangdong Provincial People's Hospital (Guangdong Academy of Medical Sciences) Southern Medical University Guangzhou Guangdong China; ^7^ Institute for Translational Brain Research, State Key Laboratory of Medical Neurobiology, MOE Frontiers Center for Brain Science, Fudan University Shanghai China; ^8^ Department of Thoracic Medical Oncology Zhejiang Cancer Hospital Hangzhou China; ^9^ Department of Oncology The Second Affiliated Hospital of Nanchang University Nanchang Jiangxi China; ^10^ Department of Thoracic Oncology Clinical Oncology School of Fujian Medical University, Fujian Cancer Hospital Fuzhou China; ^11^ Department of Neurosurgical Oncology Clinical Oncology School of Fujian Medical University, Fujian Cancer Hospital Fuzhou China; ^12^ Department of Radiation Oncology Fujian Cancer Hospital & Fujian Medical University Cancer Hospital Fuzhou China

**Keywords:** expert consensus, leptomeningeal metastases, lung cancer

## Abstract

Lung cancer patients with leptomeningeal metastases (LMs) suffer from severe clinical symptoms, poor quality of life, narrow diagnostic and therapeutic time windows, low response to standard treatments, short survival periods, and poor prognoses. At present, there is a lack of precise diagnosis and treatment pathways and relevant consensus for LMs of lung cancer. In order to better guide the clinical diagnosis and treatment of LMs of lung cancer in an early, reasonable, and safe way, the experts of the Neurological Tumor Specialist Committee of the Chinese Society of Clinical Oncology (CSCO) have formulated this consensus based on the actual diagnosis and treatment of LMs of lung cancer in China, with reference to the latest research data, relevant guidelines and consensus, and the experts' clinical experience, as well as the results of experts' polling on the pre‐set issues of LMs. The consensus focuses on diagnosing LMs, stratified management, efficacy evaluation systems, treatment strategies, common complications, and palliative care. It gives recommendations in each of the five aspects to provide clinicians with suggestions and references for diagnosing and treating LMs in lung cancer.

## Graphical abstract

This Chinese expert consensus addresses leptomeningeal metastases (LM) in lung cancer, emphasizing early MRI‐enhanced imaging and CSF cytology for diagnosis. It recommends stratified management using LM‐molGPA and targeted therapies (e.g., third‐generation EGFR‐TKIs). Intrathecal pemetrexed and Ommaya reservoirs are prioritized for treatment and palliative care. Though improvements are needed, the RANO LM system is endorsed for efficacy evaluation.

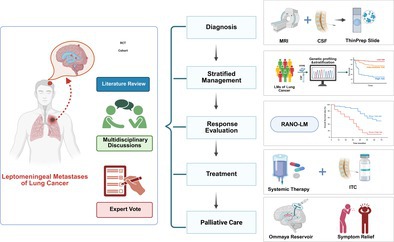

## 1 Introduction

Leptomeningeal metastases (LMs) are serious complications that occur when tumor cells spread to the leptomeninges of the brain and spinal cord, most commonly due to lung cancer. LMs occur in 3%–5% of patients with non‐small cell lung cancer (NSCLC), and this rate increases to 9% in patients with epidermal growth factor receptor (EGFR) mutations [1]. The prognosis for patients with LMs is poor, with median overall survival (OS) around 3.6–11 months [2].

Due to the absence of clear diagnostic and treatment guidelines for LMs in lung cancer, the expert group from the Neurological Tumor Specialist Committee of the Chinese Society of Clinical Oncology (CSCO) developed a consensus. This consensus was registered in December 2023 on the Practice guideline REgistration transPAREncy platform (PREPARE) with the registration number PREPARE‐2023CN971. The literature was extensively reviewed using Pubmed, Embase, China National Knowledge Infrastructure (CNKI), and Wanfang Data up to May 2024 and updated in February 2025. The consensus involved 106 experts who voted on 25 pre‐set questions, leading to a calculation of expert agreement levels. The quality of the literature and the classification of the level of evidence in this consensus were based on the European Society for Medical Oncology (ESMO) Consensus Evidence System (Table S1) [3] and the evidence categories of the CSCO Guidelines (Table S2) [4]. Regarding converting evidence into recommendations, the expert group mainly referred to the criteria for the CSCO guideline's recommendation level [4] and modified the grading of recommendations accordingly (Table 1). Finally, the strength of recommendation was categorized into three levels: Level I (strong), Level II (moderate), and Level III (weak).

**TABLE 1:** Recommendation levels used in this consensus.

Recommended level	Standard
Class I recommendations	Supported by high‐quality research evidence and a high degree of expert consensus can be used as a strong recommendation Classes I and II evidence; expert consensus: 80%–100% agreement by vote (i.e., proportion of experts choosing “agree”)
Class II recommendations	Supported by high‐quality research evidence, generally a consistent degree of expert consensus as well as better evidence, a high degree of expert consensus can be used as a moderate recommendation Classes I and II evidence, degree of expert consensus: 60%–79% agreement by vote Classes III, IV, and V evidence; expert consensus level: 80%–100% agreement
Class III recommendation	Supported by better evidence, relatively insufficient evidence from evidence‐based medicine, and largely unanimous expert consensus as a weak recommendation for Class III, IV, and V evidence; expert consensus: 60%–79% agreement by vote

The consensus covers five key areas: diagnosis, management stratification, efficacy evaluation, treatment options, and palliative care for LMs in lung cancer, offering clinicians essential guidance.

## 2 Diagnosis of Leptomeningeal Metastases

**Consensus 1:** LMs and parenchymal brain metastases (BMs) should be treated differently and managed as distinct diseases.

**Level of recommendation:** Level II, with 99% experts agreeing.

Leptomeningeal metastases involve the spread of tumor cells in the cerebrospinal fluid (CSF) and are commonly seen in breast cancer, lung cancer, and melanoma [5, 6, 7]. Parenchymal brain metastases (BMs) involve direct invasion of tumor cells into the brain tissue. Clinically, the symptoms of LMs are usually more widespread and non‐specific [6], whereas symptoms of BMs depend on the tumor's location and may include neurological dysfunction [8]. Treatment of LMs is usually based on systemic therapy, including CSF drainage and intrathecal chemotherapy (ITC) [7, 9, 10], while BMs may require localized therapy, such as surgical resection and radiotherapy [8, 11]. In terms of prognosis, LMs are usually poor [9], whereas BMs depend on several factors and may have a better prognosis if localized control is possible [8].

**Consensus 2:** LMs should be highly suspected when patients with lung cancer present with neuropsychiatric symptoms such as headache, nausea and vomiting, gait difficulties, visual disturbances, and mental status changes.

**Level of recommendation**: Level II, with 99% experts agreeing.

Symptoms of patients with LMs are often complicated and varied due to different sites of tumor cell invasion and lack of specificity, and sometimes, it is difficult to distinguish them from symptoms caused by BMs and toxic side effects of treatment. Common clinical manifestations occurring in more than 10% of patients include involvement of the cerebral hemispheric meninges (~50%), typically presenting as dizziness, headache, nausea, and vomiting; cranial nerve involvement (~40%), often presenting as visual disturbances, diplopia, hearing impairment, and visual field defects; and spinal cord and spinal nerve root involvement (exceeding 60%), commonly presenting as limb weakness, sensory abnormalities, and sensory ataxia [8, 12]. In a study examining 150 cases of solid tumors with LMs, the most frequently observed clinical signs and symptoms were headache (39%), nausea and vomiting (25%), and lower limb weakness (21%) [13].

**Consensus 3:** For patients with clinical suspicion of LMs, diagnostic imaging is preferred to magnetic resonance imaging (MRI) of the head with scan + enhancement, with a preferred field strength of 3.0 T, and 3D volumetric thin‐slice scanning, at least 3 mm slice thickness, and reconstruction of the coronal, sagittal, and transverse planes is recommended: Focus on T1‐weighted enhancement, T2 FLAIR, and T2 FLAIR enhancement images.

**Level of recommendation:** Level II, with 97% experts agreeing.

Computed tomography (CT) has a sensitivity of 23%–38% for LMs, whereas MRI‐enhanced scans have a sensitivity of 70%–87% and a specificity of 75%–94% [14, 15]. LMs usually appear on MRI as abnormal enhancement of the meninges, including nodular, linear, bowed, focal, or diffuse enhancement [5]. 3D cranial MRI Volumetric imaging (3D‐BRAVO) enhancement scans can reduce the missed diagnosis of small lesions [16]. T1‐weighted imaging sequences are the basis of conventional MRI enhancement, but vascular enhancement may affect the lesion display; enhancement of T2‐FLAIR imaging has a flow void, which improves the detection rate of cortical, paraventricular, and leptomeningeal lesions. The study showed that the sensitivity of 3D T1‐weighted enhanced, 2D‐weighted enhanced, and 2D FLAIR‐enhanced images for LMs was 71.4%, 71.4%, and 82.9%, respectively [17]. In another study, the sensitivity of FLAIR imaging for LMs was 34% and 66% for T1‐weighted enhancement [18].

**Consensus 4:** A sufficient volume of CSF (at least 10 mL) should be obtained, and the CSF should be processed within 30 min after sampling. Smears are best obtained immediately after sampling. If the conditions for disposing of the CSF are insufficient, consider immediate fixation after sampling.

**Level of recommendation:** Level II, with 99% of experts agreeing.

Finding cancer cells in CSF is currently the gold standard for LMs diagnosis, and the commonly used test is the ThinPrep cytologic test (TCT), which has a high specificity (> 95%) but a sensitivity of only about 45%–90%. The positive rate of CSF cytology correlates with the number of samples sent, the specimen volume, and the testing time. Previous reports suggest the first lumbar puncture CSF cytology is only 50% positive. However, the positive rate increases to 75%–85% with repeated samples, and subsequent increases in the number of sampling have failed to increase the positive rate [19]. Previous studies have shown that cells in the CSF begin to degrade and decrease 30 min after lumbar puncture [20]. After 2 h of lumbar puncture, cells decreased to less than 60% [21]. Other studies have suggested that adding a fixative is one of the methods that can reduce the loss of CSF cells [20]. Literature reports that CSF delivered in 2–5 mL has a positive rate of 30.5%, and more than 5 mL has a positive rate of 55.5% [15, 22].

**Consensus 5:** Although LMs are highly suspected clinically, imaging and CSF cytology are negative, and leptomeningeal biopsy can be considered.

**Level of recommendation:** Level II, with 77% experts agreeing.

Leptomeningeal pathology is also the gold standard for the diagnosis of LMs. However, it is rarely used in clinical practice, mainly because of the limitations of complex biopsy site selection, the small number of samples, and the high risk of biopsy [22]. Multiple CSF sampling can improve the positive rate of LMs diagnosis, and the literature reports that there is a risk of induced cerebral herniation in patients with severe intracranial hypertension, and more than three examinations do not help improve the positive rate of diagnosis [15]. Therefore, if imaging and CSF cytology are negative on multiple testings but clinically highly suspicious for LMs, a leptomeningeal biopsy may be considered, which requires a multidisciplinary discussion and is performed by a neurosurgeon.

## 3 Stratified Leptomeningeal Metastases

**Consensus 6:** The CSF is positive for cytology, and even if there are no symptoms associated with LMs, immediate intervention treatment is recommended.

**Level of recommendation:** Level II, with 79% experts agreeing.

A study demonstrated that patients with CSF cytology‐positive LMs experienced a worse prognosis according to the European Association for Neuro‐Oncology and European Society of Medical Oncology (EANO‐ESMO) criteria [7]. Survival was shorter for patients with type I (diagnosed by CSF cytology/leptomeningeal histopathology) LMs than for those with type II (no histocytologic evidence, only imaging or clinical symptoms) LMs [9]. However, another study indicated that the OS was similar for type I and type II LMs in NSCLC [23]. This suggests that while positive CSF cytology may indicate a poorer prognosis, its significance in lung cancer remains unclear. Additionally, the study included fewer patients with asymptomatic LMs, leading to insufficient data regarding the necessity for immediate treatment in these cases. Nevertheless, given that patients with positive CSF cytology tend to have rapid disease progression and a poor prognosis, prompt intervention is recommended for these patients.

**Consensus 7:** Lung cancer with LMs has great heterogeneity, and prognostic stratification should be performed. The LM‐molGPA classification recommended by EANO‐ESMO can be considered.

**Level of recommendation:** If, with 99% of experts agreeing.

The diagnosis of LMs is based on clinical symptoms and signs, imaging, CSF cytology, and leptomeningeal histopathology. The clinical and pathological characteristics of patients with different LMs also differ, suggesting that this group is heterogeneous. EANO‐ESMO classifies patients with LMs from solid tumors into type I and type II, subdividing each type into five main subtypes based on MRI manifestations [7]. Prognostic analysis of patients with LMs of solid tumors showed that patients with type II had significantly better survival than those with type I. Among the type II population, patients with nodular lesions on MRI had a worse prognosis [9].

A study combined information on molecular alterations to establish a prognostic model for LMs of lung cancer. In this model, EGFR or anaplastic lymphoma kinase (ALK) gene alterations, Karnofsky performance status (KPS) score, and the presence or absence of extracranial metastases are independent risk factors for the prognosis of LMs of lung cancer. Subsequently, the hazard ratio and P value are combined for weighted scoring. The total score of these three variables is the LM‐molGPA score, which is divided into high‐risk, intermediate‐risk, and low‐risk groups. The survival times of the three groups are 0.3, 3.5, and 15.9 months, respectively [24].

**Consensus 8:** Positive CSF circulating tumor DNA (ctDNA) is an independent prognostic factor in patients with LMs.

**Level of recommendation:** Level II, with 88% experts agreeing.

A CSF genomic profiling study of NSCLC central nervous system (CNS) metastases found little difference in survival between patients with type I and type II LMs in NSCLC. However, another result from this study suggested that in patients with LMs from NSCLC, the median OS time of the CSF ctDNA‐positive population was significantly shorter than the median OS time of the CSF ctDNA‐negative population [23], and subsequent updated data suggest similar findings [25]. More targeted treatment based on ctDNA genotyping should be explored in the future.

**Consensus 9**: The detection of RB1 and CDKN2A alterations in CSF indicates a poor prognosis.

**Level of recommendation:** Level II, with 91% experts agreeing.

A retrospective study of patients with LMs who had a sensitive EGFR mutant showed that intracranial PFS was shorter in patients receiving osimertinib if a genetic alteration in the CDK4 or CDKN2A gene was also detected in the CSF [26]. In addition, a study's results showed significant differences between the CSF gene profiles of patients with LMs and extracranial specimens; LMs with a CDKN2A/CDKN2B deletion detected in the CSF had significantly poorer survival [27]. Meanwhile, a CSF gene profiling study showed that TP53, CDKN2A, MYC, and RB1 gene alterations detected in CSF were associated with a worse prognosis in univariate analysis. However, only RB1 gene alterations were associated with poor survival in multivariate analysis [23]. In subsequent analyses, the investigators further confirmed that RB1 gene alterations remained an independent risk factor for poor OS prognosis [25].

## 4 Leptomeningeal Metastases Response Evaluation System

**Consensus 10:** It is recommended that prospective clinical trials use OS as the primary endpoint.

**Level of recommendation:** Level II, with 91% experts agreeing.

The early efficacy evaluation system of LMs was based on improving symptoms and signs and improving CSF cytology and biochemical indexes to evaluate efficacy [28]. In 1993, the Eastern Cooperative Oncology Group (ECOG) conducted a prospective study of 59 cases with LMs receiving intrathecal treatment; the three factors of symptoms and signs, CSF result, and CT imaging evaluation were comprehensively scored for the first time [29]. In 2017, the Response Assessment in Neuro‐Oncology Leptomeningeal Metastases (RANO LM) Working Group and the RANO Neurological Assessment Working Group jointly launched the RANO efficacy evaluation system, which, for the first time, standardized the definition of response and included symptom scoring scales, CSF cytology testing, and MRI scoring scales [5]. In practice, even with the standardized evaluation process of scale scores brought about by RANO, the stable assessment of the short‐term efficacy of LMs, such as progression‐free survival (PFS) and objective response rate (ORR), is still tricky. The reproducibility of the imaging assessment is poor, especially for linear enhancement and cranial nerve enhancement [30]. The inconsistency of imaging assessment has led to significant variations in short‐term efficacy evaluations. In the BLOOM study, for example, the blinded independent central review (BICR) evaluation of LMs efficacy evaluated 32% of patients with a complete response (CR) rate, compared with only 2% of patients with a CR rate in the investigator's evaluation [31]. In another meta‐analysis of NSCLC LMs, PFS in seven studies ranged from 3.7 to 13.7 months, with five of these studies reporting OS data with a median OS of 11.0–18.8 months, illustrating the precarious nature of the use of short‐term efficacy in LMs patients [32].

**Consensus 11:** Currently, there are multiple efficacy evaluation systems in parallel, and the RANO system is mainly used in clinical settings. However, it has consistency biases and needs further improvement and perfecting.

**Level of recommendation:** Level II, all experts (100%) agree.

After the RANO evaluation system was introduced in 2017, many clinical studies began using it to evaluate efficacy. However, this standard is still a consensus rather than a guideline, and because the scale is difficult to operate, the evaluation is time‐consuming and inconsistent [30]. As of October 2023, among the 19 prospective studies on LMs that have been published and the 84 prospective studies currently underway registered on the ClinicalTrials website, the following efficacy evaluation systems can be found: signs and symptoms alone, imaging alone, CSF alone; a two‐factor evaluation system of signs and symptoms combined with KPS score; a three‐factor comprehensive efficacy evaluation system of RANO or a modified version of signs and symptoms combined with imaging and CSF; and multi‐system evaluation systems such as metabolomics response. Therefore, there are multiple efficacy evaluation systems in parallel, with the RANO system being the mainstay in clinical practice [33]. However, consistency biases in its use still need to be further improved and perfected. Depending on the research objective, targeted treatment studies often use the Response Evaluation Criteria In Solid Tumors (RECIST) to evaluate the comprehensive assessment of extracranial and brain parenchymal combined with RANO LM imaging manifestations. In contrast, studies of intrathecal treatment are evaluated using the symptoms, signs, and KPS score [34] or the RANO system.

## 5 Treatment of Leptomeningeal Metastases

### 5.1 Intrathecal Chemotherapy (ITC)

**Consensus 12:** ITC is an important palliative treatment for LMs and, as one of the cornerstones of the treatment program, can significantly help improve patient symptoms and quality of life.

**Level of recommendation:** Level II, with 89% experts agreeing.

ITC is a treatment method that directly infuses chemotherapy drugs into the CSF, bypassing the blood–brain barrier (BBB) and allowing the drugs to target tumor cells with LMs, thereby improving treatment outcomes [10].

Most current prospective studies on ITC for lung cancer are pilot studies with small sample sizes. Drawing on the experience of the DEPOSEIN study in breast cancer, which was the first phase III randomized controlled trial to evaluate the effects of systemic therapy combined with ITC in patients with LMs from breast cancer, the results showed that the combination treatment group was superior to the systemic treatment group in terms of clinical improvement, radiographic improvement, and CSF cytology remission. In addition, the median LM‐PFS of the combined treatment group was 3.8 months, better than the 2.2 months of the systemic treatment group [35].

**Consensus 13:** The selection of intrathecal chemotherapeutic agents for NSCLC LMs can include pemetrexed, methotrexate, thiotepa, and cytarabine. Pemetrexed is recommended as the preferred intrathecal chemotherapeutic agent. The efficacy of ITC with pemetrexed is not compromised after previous systemic chemotherapy with pemetrexed.

**Level of recommendation:** Level II, with 92% experts agreeing.

For LMs, researchers have studied the clinical efficacy of ITC with various drugs, including pemetrexed, methotrexate, thiotepa, and cytarabine. Pemetrexed and methotrexate are the most commonly used intrathecal drugs in NSCLC [36, 37].

One retrospective study showed that median intracranial PFS was 7.3 months for patients receiving intrathecal methotrexate and 8 months for patients receiving intrathecal pemetrexed [38]. Methotrexate inhibits tumor cell proliferation by inhibiting DNA and RNA synthesis, while pemetrexed blocks DNA replication and cell division in tumor cells by inhibiting several key enzymes. Pemetrexed has been shown to have superior antitumor activity in systemic chemotherapy of NSCLC [39]. The BBB limits the concentration of chemotherapeutic drugs in the brain. However, a study showed that intrathecal pemetrexed can significantly benefit patients even after they have failed multiple lines of prior treatment, including systemic chemotherapy with pemetrexed [34].

**Consensus 14:** The recommended starting dose of ITC with pemetrexed is 10–50 mg, 1–2 times a week, with multiple administrations. The pretreatment is the same as intravenous pemetrexed treatment. The specific dose and course of treatment are individualized according to the patient's condition.

**Level of recommendation:** Level I, with 99% experts agreeing.

There is no uniform standard for the dosage, course of treatment, and frequency of administration of intrathecal pemetrexed, and clinical trial designs vary. In one study, 11 patients were given intrathecal pemetrexed twice weekly at a recommended dose of 10 mg. The ORR was 36%, the disease control rate (DCR) was 64%, the median intracranial PFS was 2.5 months, and the OS was 3.8 months [40]. Thirty patients were treated with induction treatment twice weekly, followed by consolidation treatment every 3 months until progressive disease was observed. The recommended dose was 50 mg, and the ORR was 84.6%, with a median OS of 9 months [34]. Another retrospective study used an incremental dose approach, with a dose range of 10–50 mg, a median intracranial PFS of 8 months, and a median OS of 9.6 months [38].

### 5.2 Targeted Therapies

**Consensus 15:** Initial treatment of patients with LMs: The typical dose is recommended. Third‐generation EGFR tyrosine kinase inhibitors (EGFR‐TKIs) are preferred for those harboring an EGFR mutation, and second‐ or third‐generation ALK tyrosine kinase inhibitors (ALK‐TKIs) are preferred for those harboring an ALK fusion.

**Level of recommendation:** Level I, with 97% experts agreeing.

The occurrence of LMs is closely related to the molecular subtype of primary cancer. Studies have shown that the incidence of LMs in patients with EGFR‐mutant lung cancer is significantly higher than that in patients with wild‐type EGFR (9.4% vs. 1.7%) [41]. Targeted therapy is beneficial for these patients [42, 43]. Third‐generation EGFR‐TKIs such as osimertinib have demonstrated superior efficacy in treating patients with EGFR‐mutant NSCLC with LMs, compared to first‐ and second‐generation EGFR‐TKIs [44]. Osimertinib significantly reduces the rate of CNS progression and the risk of new BMs, as well as prolongs OS. Previous studies have confirmed the advantages of third‐generation EGFR‐TKIs in treating EGFR‐mutant patients with LMs [45, 46, 47].

Compared to the efficacy results of previous studies on LMs, the efficacy of the standard dose of osimertinib 80 mg is not inferior to that of the doubled dose of 160 mg [2, 31, 48]. At present, there is a lack of results from prospective studies that compare different doses of osimertinib head‐to‐head. A meta‐analysis showed that 353 patients with EGFR‐mutant NSCLC with LMs received 80 mg osimertinib, with an ORR of 47%, compared with 37% in the 160 mg treatment group [32]. Current evidence does not support the superiority of double‐dose third‐generation EGFR‐TKI treatment over standard doses.

About 10% of NSCLC patients with ALK fusions develop LMs [49], and up to 40% have CNS metastases at the time of diagnosis [50]. Although the first‐generation ALK inhibitor crizotinib has limited ability to penetrate the CNS [51], second‐ and third‐generation drugs such as alectinib, brigatinib, ceritinib, and lorlatinib can effectively penetrate the BBB and have a better therapeutic effect on CNS lesions [52]. In the CROWN trial, lorlatinib showed longer PFS and a higher intracranial ORR than crizotinib [53].

**Consensus 16:** Post‐treatment of TKI resistance in LMs 1: There are significant differences genome profiling between the CSF and the primary tumor in patients with LMs, CSF genetic testing is strongly recommended to clarify the mechanism of drug resistance.

**Level of recommendation:** Level II, with 97% experts agreeing.

Studies have found that CSF cfDNA can effectively reflect the molecular differences between extracranial and LMs lesions. In 28 patients with NSCLC, CSF cfDNA revealed a unique genetic profile, especially multiple copy number variations, compared with plasma and primary tissue [54]. Another study of 33 NSCLC patients with LMs showed that 161 point mutations detected in CSF cfDNA included driver mutations in all patients, and the number was much higher than that in peripheral blood cfDNA [55]. Therefore, CSF cfDNA is the most representative liquid biopsy specimen for LMs in EGFR mutant NSCLC. A molecular graded prognostic assessment (molGPA) model incorporates EGFR and ALK alterations as prognostic factors, which helps to distinguish the response of different subtypes to treatment [24].

**Consensus 17:** Post‐treatment of TKI resistance in LMs 2: Resistance mechanisms are clear, drugs are available, and individualized treatment based on ITC is recommended.

**Level of recommendation:** Level II, with 93% experts agreeing.

CSF cfDNA can reveal the genetic characteristics of LMs and guide subsequent treatment. In a study, 22 patients who developed LMs after resistance to osimertinib and received targeted treatment matched the results of CSF genetic testing and had a median PFS of 3.2 months and a median OS of 7.2 months [56]. ITC is one of the effective treatments for NSCLC with LMs [57]. Intrathecal pemetrexed shows good application prospects in NSCLC patients with LMs who have progressed after EGFR‐TKI treatment. In a phase I study, the ORR and DCR were 31% and 54%, respectively [40]. In another trial, 30 patients showed a clinical response rate of 84.6%, and the median OS was 9 months [34].

**Consensus 18:** Post‐treatment of TKI resistance in LMs 3: The mechanism of drug resistance is unclear. Other systemic treatments are recommended based on ITC, and participation in clinical research is encouraged.

**Level of recommendation:** Level II, with 99% experts agreeing.

Systemic chemotherapy is the treatment of choice for NSCLC patients with LMs without driver mutations that have spread systemically, as studies have shown that it is an independent predictor of survival [58]. A clinical trial including 132 patients showed that for NSCLC patients with LMs after failure of EGFR‐TKI treatment, without exploring the mechanism of drug resistance, intrathecal pemetrexed treatment can achieve significant results, with a median OS of 12 months and acceptable safety, based on continuing the foremost treatment [59].

**Consensus 19:** Post‐treatment of TKI resistance in LMs 4: For unclear resistance mechanisms, pulsed administration of first‐generation EGFR‐TKI or dose escalation of third‐generation EGFR‐TKI may be an option for treating LMs. However, high‐level evidence‐based medical evidence is lacking, and more data are needed.

**Level of recommendation:** Level III, with 98% experts agreeing.

High‐dose gefitinib [60] and a pulsatile erlotinib [61] dosing strategy may increase intracranial drug concentrations and benefit some patients with EGFR‐mutant NSCLC with LMs. The BLOOM trial showed that double the usual dose of osimertinib (160 mg/day) in patients with LMs resulted in a duration of response of 8.3 months and an ORR of 41% [31]. Another study also showed the potential clinical efficacy and safety of high‐dose furmonertinib in treating patients with LMs in EGFR‐mutant NSCLC [62].

### 5.3 Immunotherapy

**Consensus 20:** The tumor microenvironment of LMs is more immunosuppressive than the brain parenchymal metastases and is characterized by a rich infiltration of immunosuppressive cell subsets such as MDSCs, M2‐TAMs, and Tregs.

**Level of recommendation:** Level I, with 97% experts agreeing.

Research on the tumor microenvironment (TME) has focused on the cellular composition and status of the leptomeningeal microenvironment, especially immune cells and tumor cells. Usually, immune cells in the leptomeninges maintain a balance [63, 64], but in patients with LMs, this balance is disrupted, and immunosuppressive cells such as MDSCs, M2‐TAMs, and Tregs increase [65, 66]. One study found that tumor cells deprive macrophages of iron by expressing LCN2 and SLC22A17, which makes macrophages to lose their immune surveillance function [63]. Another study found that tumor cells secrete C3, which disrupts the BBB and promotes tumor cell proliferation, suggesting that C3aR can be used as a treatment target [67].

**Consensus 21:** For driver gene‐negative NSCLC with LMs, monotherapy with PD‐1/PD‐L1 inhibitors has limited efficacy.

**Level of recommendation:** Level II, with 94% experts agreeing.

Immunotherapies such as PD‐1/PD‐L1 and CTLA4 monoclonal antibodies have lasting effects in a small proportion of patients with LMs. A European study showed that 19 patients with NSCLC and LMs treated with PD‐1 monoclonal antibodies had a median OS of 3.7 months [68]. In another study, 13 patients with LMs from different cancer types treated with pembrolizumab had a median OS of 4.9 months [69]. The efficacy can be partially improved by combined chemoradiotherapy or bevacizumab treatment. A retrospective study found that the median OS of patients treated with PD‐1/PD‐L1 monoclonal antibody combination therapy was 5.4 months, while that of patients treated with monoclonal antibody therapy alone was 4.0 months [70].

### 5.4 Antivascular Treatment

**Consensus 22:** The LMs have active tumor angiogenesis in the local microenvironment. Therefore, targeted combined anti‐angiogenic treatment modalities can be considered for patients with driver gene‐positive LMs.

**Level of recommendation:** Level II, with 98% experts agreeing.

Bevacizumab is a monoclonal antibody that targets vascular endothelial growth factor (VEGF) and inhibits angiogenesis and tumor growth. One trial showed that osimertinib in combination with bevacizumab was effective in treating patients with LMs of EGFR‐mutant NSCLC. The median PFS and OS were 9.3 months and 12.6 months, respectively [71]. Another study also showed that the median OS of the combination treatment group was 18.0 months, which was better than that of the targeted monotherapy group (13.7 months) [72].

#### 5.4.1 Craniospinal Irradiation

**Consensus 23:** Indications for radiotherapy: For leptomeningeal nodular lesions, precise radiotherapy of the involved field can be considered, and the use of craniospinal irradiation needs to be carefully considered.

**Level of recommendation:** Level II, with 96% experts agreeing.

Radiotherapy can effectively alleviate the symptoms of LMs, and especially for nodular lesions, precise radiotherapy can be considered. One study showed that the median OS of patients with LMs who received stereotactic radiosurgery (SRS) was higher than that of patients who did not [73]. Another study showed that palliative radiotherapy can effectively stabilize or improve neurological symptoms [74]. However, craniospinal irradiation (CSI) may cause toxicity, and its use should be carefully considered [75]. Proton CSI has shown potential advantages in the treatment of LMs. Compared with traditional photon radiotherapy, proton CSI has prolonged PFS and OS in the CNS metastases, with no significant difference in toxic reactions [76].

## 6 Common Complications of Leptomeningeal Metastases and Palliative Care

**Consensus 24:** ITC is recommended for patients with LMs. The administration route can be via the Ommaya reservoir, an intrathecal infusion pump in the lumbar spine, or repeated lumbar punctures. The Ommaya reservoir is preferred.

**Level of recommendation:** Level II, with 97% experts agreeing.

ITC via the Ommaya reservoir has been successfully used in the treatment of LMs [77, 78, 79, 80]. The main routes of administration of ITC include direct injection of the Ommaya capsule into the ventricle, injection into the lumbar dura through a lumbar puncture, or intrathecal injection through a lumbar Ommaya pump [81, 82]. Compared with intrathecal injection through a lumbar puncture, intrathecal injection into the ventricle allows the drug to be evenly distributed throughout the CNS with the circulation of CSF, and the concentration can reach 10 times that of the same dose administered through a lumbar puncture [83], with better survival benefits [84, 85]. Compared with repeated lumbar puncture for drug administration, administration through the Ommaya reservoir is safer and more convenient. CSF drainage can be performed according to intracranial pressure to improve symptoms of intracranial hypertension promptly. It is also convenient for repeated CSF sampling to understand the dynamic changes in drug concentration and tumor biomarkers [86, 87, 88].

**Consensus 25:** CSF shunting or Ommaya reservoir implantation is strongly recommended for LMs with refractory intracranial hypertension or hydrocephalus.

**Level of recommendation:** Level II, with 99% experts agreeing.

CSF shunting in patients with LMs from lung cancer with severe headache, refractory cranial hypertension, or hydrocephalus. CSF shunting can quickly relieve symptoms, help improve the quality of life, and facilitate subsequent targeted treatment [89]. Shunting can be performed using a ventriculoperitoneal shunt, a lumboperitoneal shunt, or continuous external drainage via an Ommaya reservoir implantation [90, 91]. Three factors are associated with a better prognosis after shunting: the availability of suitable targeted treatment drugs, a good KPS score, and reasonable control of extracranial lesions. These three criteria can be the best indications for CSF shunting in patients with LMs [92]. Shunting is not recommended for patients in generally poor condition and/or with a KPS of less than 30 because shunting surgery cannot improve the prognosis of terminal‐stage patients [93].

## Author Contributions

Gen Lin, Yongsheng Wang, Tao Xin, Ding Zhang, Qiuyu Zhang, Yangsi Li, Yudan Chi, Yun Fan, Anwen Liu were responsible for the design of this article. Haipeng Xu, Liang Shi, Longfeng Zhang, Qian Miao, Xiaobin Zheng, Lijun Li, Kaijia Zhou, Qiwei Yao, Zihua Zou, Kang Miao, Yaping Hong, Gen Lin drafted this manuscript. All authors reviewed the final version of the manuscript.

## Conflicts of Interest

The authors declare no conflicts of interest.

## Supporting information


**Data S1** Supplementary Information.

## Data Availability

The data supporting the findings of this study are available from the corresponding author upon request.

## Appendix A

### Expert Consensus Drafting Group

**Haipeng Xu:** Department of Thoracic Oncology, Clinical Oncology School of Fujian Medical University, Fujian Cancer Hospital, Fuzhou, China.

**Liang Shi:** Department of Medical Oncology, Beijing Tuberculosis and Thoracic Tumor Research Institute, Beijing Chest Hospital, Capital Medical University, Beijing 101 149, China.

**Longfeng Zhang:** Department of Thoracic Oncology, Clinical Oncology School of Fujian Medical University, Fujian Cancer Hospital, Fuzhou, China.

**Qian Miao:** Department of Thoracic Oncology, Clinical Oncology School of Fujian Medical University, Fujian Cancer Hospital, Fuzhou, China.

**Xiaobin Zheng:** Department of Medical Oncology, Beijing Tuberculosis and Thoracic Tumor Research Institute, Beijing Chest Hospital, Capital Medical University, Beijing 101 149, China.

**Lijun Li:** Institute of Immunotherapy, The School of Basic Medical Sciences of Fujian Medical University, Fuzhou, China.

**Kaijia Zhou:** Department of Neurosurgical Oncology, Clinical Oncology School of Fujian Medical University, Fujian Cancer Hospital, Fuzhou, China.

**Qiwei Yao:** Department of Radiation Oncology, Fujian Cancer Hospital & Fujian Medical University Cancer Hospital, Fuzhou 350 014, China.

**Zihua Zou:** Department of Thoracic Oncology, Clinical Oncology School of Fujian Medical University, Fujian Cancer Hospital, Fuzhou, China.

**Kang Miao:** Department of Thoracic Oncology, Clinical Oncology School of Fujian Medical University, Fujian Cancer Hospital, Fuzhou, China.

**Yaping Hong:** Department of Thoracic Oncology, Clinical Oncology School of Fujian Medical University, Fujian Cancer Hospital, Fuzhou, China.

### Other Voting Experts

**Yu Yao**: Department of Medical Oncology, The First Affiliated Hospital of Xi'an Jiaotong University, Xi'an, Shaanxi, China.

**Baosong Xie**: Department of Respiratory and Critical Care Medicine, Fuzhou University Affiliated Provincial Hospital, Fujian Provincial Hospital, Fuzhou, 350 001, China.

**Chengzhi Zhou**: The First Affiliated Hospital of Guangzhou Medical University, National Center for Respiratory Medicine, National Clinical Research Center for Respiratory Disease, State Key Laboratory of Respiratory Disease, Guangzhou Institute of Respiratory Health, Guangzhou, 510 000, China.

**Meijuan Huang**, Division of Thoracic Tumor Multimodality Treatment and Department of Medical Oncology, Cancer Center, West China Hospital, Sichuan University.

**Baoshan Cao**: Cancer center, Peking University Third Hospital/Department of Medical Oncology and Radiation Sickness, Peking University Third Hospital, Beijing, China.

**Jie Lin**: Department of Medical Oncology, The Second Affiliated Hospital of Kunming Medical University, 374 Dianmian Avenue, Wuhua, Kunming, Yunnan 650 101, People's Republic of China.

**Tianqing Chu**: Department of Pulmonary, Shanghai Jiao Tong University School of Medicine, Shanghai Chest Hospital, Shanghai, China.

**Rongbo Lin**: Department of Gastrointestinal Oncology, Clinical Oncology School of Fujian Medical University, Fujian Cancer Hospital, Fuzhou, China.

**Zongyang Yu**: Pulmonary and Critical Care Medicine, The 900th Hospital of the Joint Logistic Support Force, PLA, Fuzhou, China.

**Wenyuan Ye**: Chinese and Western Medicine Integrated Oncology Department, Fuzhou First General Hospital, Fuzhou, China.

**Qing Bu**: Department of Medical Oncology, The First Affiliated Hospital of Guangxi Medical University, Nanning, China.

**Ding Zhang**: Pulmonary and Critical Care Medicine, Huashan Hospital, Fudan University, Shanghai, China.

**Bo Shen**: Department of Oncology, The Affiliated Cancer Hospital of Nanjing Medical University & Jiangsu Cancer Hospital & Jiangsu Institute of Cancer Research, Nanjing, Jiangsu Province, China.

**Kai Yin**: Guangdong Lung Cancer Institute, Guangdong Provincial People's Hospital (Guangdong Academy of Medical Sciences), Southern Medical University, Guangzhou, Guangdong, China.

**Chuanhao Tang**: Peking University International Hospital, Life Park Road No. 1, Life Science Park of Zhong Guancun, Changping District, Beijing 102206, China.

**Huijuan Wang**: Department of Medical Oncology, The Affiliated Cancer Hospital of Zhengzhou University/Henan Cancer Hospital, 127 Dongming Rd., Zhengzhou 450 003, China.

**Zhijie Wang**: State Key Laboratory of Molecular Oncology, CAMS Key Laboratory of Translational Research on Lung Cancer, Department of Medical Oncology, National Cancer Center/National Clinical Research Center for Cancer/Cancer Hospital, Chinese Academy of Medical Sciences Peking Union Medical College, Beijing, 100 021, China.

**Wenxian Wang**: Department of Chemotherapy, Chinese Academy of Sciences University Cancer Hospital (Zhejiang Cancer Hospital), Hangzhou, China.

**Bo Jin**: Department of Medical Oncology, The First Hospital of China Medical University, Shenyang, China.

**Yintao Li**: Department of Respiratory Oncology, Shandong Cancer Hospital and Institute, Shandong First Medical University, and Shandong Academy of Medical Sciences, Jinan, Shandong, China.

**Li Li**: Department of Respiratory Diseases, Daping Hospital, Army Medical University (Third Military Medical University), Chongqing, China.

**Yan Wang**: Department of Medical Oncology, National Cancer Center/National Clinical Research Center for Cancer/Cancer Hospital, Chinese Academy of Medical Sciences Peking Union Medical College, Beijing, China.

**Qian Chu**: Department of Oncology, Tongji Hospital, Tongji Medical College, Huazhong University of Science and Technology, Wuhan, China.

**Jun Wang**: Department of Oncology, The First Affiliated Hospital of Shandong First Medical University & Shandong Provincial Qianfoshan Hospital, Jinan, China.

**Panwen Tian**: Department of Pulmonary and Critical Care Medicine, Lung Cancer Center, West China Hospital, Sichuan University, Precision Medicine Key Laboratory of Sichuan Province, Chengdu, Sichuan, China.

**Fang Wu**: Department of Oncology, The Second Xiangya Hospital, Central South University, Changsha, China.

**Xiaojun Lv**: Department of Oncology, Xiamen Humanity Hospital, Xiamen, China.

**Shengxiang Ren**: Department of Medical Oncology, Shanghai Pulmonary Hospital, School of Medicine, Tongji University, Shanghai 200 433, China.

**Guixian Zhao**: Department of Neurology, Huashan Hospital Shanghai Medical College, Fudan University, 12 Wulumuqi Zhong Road, Shanghai 200 040, China.

**Jun Zhao**: Key Laboratory of Carcinogenesis and Translational Research (Ministry of Education), name of department/division, Peking University Cancer Hospital & Institute, Beijing 100 142, China.

**Chuanqian Huang**: Department of Oncology, Ningde Municipal Hospital of Ningde Normal University, Ningde, China.

**Xiuyu Cai**: Department of VIP Inpatient, Sun Yet‐Sen University Cancer Center, Guangdong Provincial Clinical Research Center for Cancer, State Key Laboratory of Oncology in South China, Collaborative Innovation Center for Cancer Medicine, 651 Dongfeng Road East, Guangzhou, China.

**Chunxia Su**: Department of Oncology, Shanghai Pulmonary Hospital, School of Medicine, Tongji University, Shanghai, China.

**Zhigang Liu**: Cancer Center, The Tenth Affiliated Hospital of Southern Medical University, Dongguan People's Hospital, Guangdong Province, 523 000, China.

**Bin Xu**: Cancer Center, Wuhan University Renmin Hospital Wuhan, China.

**Tongjian Cui**: Department of Oncology, Fuzhou University Affiliated Provincial Hospital, Fuzhou, China.

**Diansheng Zhong**: Department of Thoracic Oncology, Tianjin Medical University Cancer Institute and Hospital, Tianjin, 300 060, People's Republic of China.

**Wenbin Qiu**: Department of Oncology, Fuding Hospital, Fuding, China.

**Bin Wei**: School of Life Sciences, Shanghai University, Shanghai 200 444, China.

**Bingqing Luo**: Department of Respiratory Oncology, The Second Affiliated Hospital of Xiamen Medical College, Xiamen, China.

**Zhentian Liu**: Department of Thoracic Oncology, Jiangxi Cancer Hospital, Nanchang, China.

**Huita Wu**: Department of Oncology, Zhongshan Hospital of Xiamen University, School of Medicine, Xiamen University, Xiamen, China;

School of Clinical Medicine, Fujian Medical University, Fuzhou, China.

**Chuan Xu**: Department of Oncology & Cancer Institute, Sichuan Provincial People's Hospital, University of Electronic Science and Technology of China.

**Chengbo Han**: Department of Oncology, Shengjing Hospital of China Medical University, Shenyang, China.

**Xiaolong Yan**: Department of Thoracic Surgery, Tangdu Hospital.

**Jinliang Wang**: Department of Oncology, Five medical center of Chinese PLA general hospital, Beijing, China.

**Dingzhi Huang**: Tianjin Medical University Cancer Institute and Hospital, National Clinical Research Center for Cancer; Key Laboratory of Cancer Prevention and Therapy, Tianjin;

Tianjin's Clinical Research Center for Cancer;

Department of Thoracic Oncology, Tianjin Lung Cancer Center, Tianjin Cancer Institute & Hospital, Tianjin Medical University, Tianjin, 300 060, People's Republic of China.

**Likun Chen**: Department of Medical Oncology, Sun Yat‐Sen University Cancer Center;

State Key Laboratory of Oncology in South China;

Collaborative Innovation Center for Cancer Medicine Guangzhou, China.

**Meimei Zheng**: Guangdong Lung Cancer Institute, Guangdong Provincial People's Hospital (Guangdong Academy of Medical Sciences), Southern Medical University, Guangzhou, 510 080, China.

**Lin Wu**: The Department of Thoracic Medical Oncology, Hunan Cancer Hospital/The Affiliated Cancer Hospital of Xiangya School of Medicine, Central South University, Changsha, China.

**Yang Xia**: Key Laboratory of Respiratory Disease of Zhejiang Province, Department of Respiratory and Critical Care Medicine, Second Affiliated Hospital of Zhejiang University School of Medicine, Hangzhou, China.

**Wenfeng Fang**: Department of Medical Oncology, State Key Laboratory of Oncology in South China, Guangdong Provincial Clinical Research Center for Cancer, Sun Yat‐sen University Cancer Center, Guangzhou, China.

**Chunwei Xu**: Department of Respiratory Medicine, Affiliated Jinling Hospital, Medical School of Nanjing University, Nanjing, Jiangsu 210 002, People's Republic of China.

**Zhenhua Liu**: Department of Oncology, Fuzhou University Affiliated Provincial Hospital, Fujian Provincial Hospital, Fuzhou, Fujian, China.

**Lijun Li**: Institute of Immunotherapy, Fujian Medical University, Fuzhou, China.

**Jing Wang**: Department of Cancer Center, Fujian Medical University Xiamen Humanity Hospital, Xiamen, China.

**Kaijia Zhou**: Department of Neurosurgical Oncology, Clinical Oncology.

School of Fujian Medical University, Fujian Cancer.

Hospital, Fuzhou, China.

**Jinghui Lin**: Department of Thoracic Oncology, Clinical Oncology.

School of Fujian Medical University, Fujian Cancer.

Hospital, Fuzhou, China.

**Xu Hai Peng**: Department of Thoracic Oncology, Clinical Oncology.

School of Fujian Medical University, Fujian Cancer Hospital, Fuzhou, China.

**Yijun Wang**: Department of Oncology, Zhangzhou Affiliated Hospital of Fujian Medical University, Zhangzhou, China.

**Weilin Chen**: Department of Radiation Oncology, Zhangzhou Affiliated Hospital of Fujian Medical University, Zhangzhou 363 000, China.

**Jialei Wang**: Department of Medical Oncology, Fudan University Shanghai Cancer Center, Shanghai, China.

**Anwen Liu**: Department of Oncology, The Second Affiliated Hospital of Nanchang University, Nanchang, Jiangxi, China.

**Weijian Zhang**: Department of Radiotherapy, The First Affiliated Hospital of Fujian Medical University, Fuzhou, 350 005, China;

Key Laboratory of Radiation Biology of Fujian Higher Education Institutions, The First Affiliated Hospital, Fujian;

Medical University, Fuzhou, 350 005, China.

**Jingxun Wu**: Department of Medical Oncology, The First Affiliated Hospital of Xiamen University, School of Medicine, Xiamen University, Xiamen, China.

**Zhengbo Song**: Department of Medical Oncology, Zhejiang Cancer Hospital, Hangzhou, China.

**Yueming He**: Department of Pulmonary and Critical Care Medicine, The First Hospital of Quanzhou Affiliated to Fujian Medical University, Quanzhou, China.

**Juntao Zou**: Department of Respiratory and Critical Care Medicine, The First Affiliated Hospital of Nanchang University, Nanchang, Jiangxi, China.

**Bicheng Zhang**: Cancer Center, Renmin Hospital of Wuhan University, Wuhan 430 060, China.

**Jian‐Guo Sun**: Department of Oncology, Xinqiao Hospital, Army Medical University, Chongqing, China.

**Longmei Dai**: Sanming Second Hospital, Sanming, China.

**Xiaoliang Wang**: Department of Radiotherapy Oncology, The Third Hospital of Zhangzhou, Zhangzhou, China.

**Chunxiang Li**: Oncology Department of integrated Chinese and Western medicine, The Second Affiliated Hospital of Fujian Medical University Quanzhou, China.

**Shijie Chen**: Department of Thoracic Oncology, Clinical Oncology School of Fujian Medical University, Fujian Cancer Hospital, Fuzhou, China.

**Qingyu Lin**: Department of Respiratory and Critical Care, Affiliated Hospital of Putian University, Putian, Fujian, China.

**Zhiyong Chen**: Department of Oncology, Longyan First Hospital, Affiliated to Fujian Medical University, People's Republic of China.

**Xi Shi**: Department of Oncology, Molecular Oncology Research Institute, The First Affiliated Hospital, Fujian Medical University, Fuzhou, Fujian 350 005, China.

**Jianchun Duan**: CAMS Key Laboratory of Translational Research on Lung Cancer, State Key Laboratory of Molecular Oncology, Department of Medical Oncology, National Cancer Center/National Clinical Research Center for Cancer/Cancer Hospital, Chinese Academy of Medical Sciences, Peking Union Medical College, Beijing 100 021, China.

**Dongyong Yang**: Department of Pulmonary and Critical Care Medicine, The Second Affiliated Hospital of Fujian Medical University, Center of Respiratory Medicine of Fujian Province, Quanzhou, China.

**Qiang Xie**: Department of Tumor, Fuzhou Pulmonary Hospital, Fuzhou, China.

**Shuitu Feng**: Department of Oncology, Fudan University Shanghai Cancer Center Xiamen Hospital, Xiamen, China.

**Qiang Wang**: Department of Thoracic Oncology, Clinical Oncology School of Fujian Medical University, Fujian Cancer Hospital, Fuzhou, China.

**Jie Hu**: Department of Respiratory Medicine, Shanghai Geriatric Medical Center, Zhongshan Hospital, Fudan University, Shanghai China.

**Meiqi Shi**: Department of Medical Oncology, Affiliated Cancer Hospital of Nanjing Medical University & Jiangsu Cancer Hospital & Jiangsu Institute of Cancer Research, Nanjing, China.

**Zhe Liu**: Department of Oncology, Beijing Chest Hospital, Capital Medical University, Beijing, China.

**Li Lin**: Zhangzhou Municipal Hospital of Fujian Province, 59 West Shengli Road, Zhangzhou, Fujian province, China.

**Yun Fan**: Department of Thoracic Medical Oncology, Zhejiang Cancer Hospital, Hangzhou, China.

**Qiu Xiang Zheng**: Department of Oncology, Longyan First Affiliated Hospital of Fujian Medical University.

**Xianhe Xie**: Department of oncology, Molecular Oncology Research institute, The First Affiliated Hospital, Fujian Medical University, Fuzhou, Fujian, China.

**Zhang Zhou Huang**: Department of Thoracic Oncology, Clinical Oncology School of Fujian Medical University, Fujian Cancer Hospital, Fuzhou, China.

**Dongrong Jiang**: 单位: Oncology Department of the Second Hospital of Longyan, The Second Hospital of Longyan Fujian, China.

**Jianxin Xu**: Department of Thoracic Surgery, The First Hospital of Putian, The School of Clinical Medicine, Fujian Medical University, 351 100 Putian, China.

**Shengchi Chen**: Department of Oncology, Nanping First Hospital Affiliated to Fujian Medical University, Nanping, China.

**Wang Sheng**: Department of Medical Oncology, The First Affiliated Hospital of Xiamen University, Xiamen, China.

**Guo Hui**: Department of Medical Oncology, The Second Affiliated Hospital of Xi'an Jiaotong University, Xi'an 710 004, China.

**Mingqiu Chen**: Department of Thoracic Oncology, Clinical Oncology School of Fujian Medical University, Fujian Cancer Hospital, Fuzhou, China.

**Jiefei Han**: Department of Neuro‐Oncology, Cancer Center, Beijing Tiantan Hospital, Capital Medical University, Beijing, China.

**Tongtong An**: Department of Thoracic Internal Medicine, Beijing Cancer Hospital, Beijing, China.

**Yong Fang**: Department of Medical Oncology, Sir Run Run Shaw Hospital, Zhejiang University School of Medicine, Hangzhou, Zhejiang, 310 016, China.

**Tongmei Zhang**: Medical Oncology, Beijing Chest Hospital, Capital Medical University & Beijing Tuberculosis and Thoracic Tumor Research Institute, Zone 1, No. 9, Beiguan Street, Tongzhou District, Beijing 101 149, China.
