# Supplementary material for: Chinese Expert Consensus on Leptomeningeal Metastases of Lung Cancer
Source: Thorac Cancer. 2025 Jun 8;16(11):e70088. doi: 10.1111/1759-7714.70088 (PMC12145986; doi:10.1111/1759-7714.70088)
Supplement: Supplementary file 1 — Data S1 Supplementary Information. [file TCA-16-e70088-s001.docx]

**Appendix:**

**Supplemental Table 1: The European Society for Medical Oncology (ESMO) Level of Evidence (2022 Edition)**

| Level of evidence | Define |
| --- | --- |
| I | Evidence from at least one large-scale randomized controlled trial of good methodological quality (with low likelihood of bias) or a meta-analysis of multiple well-executed randomized trials without non-homogeneity |
| II | Small randomized trials or large randomized trials with suspected bias (poor methodological quality), or meta-analyses of these trials or multiple trials with non-homogeneity |
| III | Prospective cohort study |
| IV | Retrospective cohort studies or case-control studies |
| V | Studies without control groups, case reports, expert opinion |

**Supplemental Table 2. Categories of evidence from the Chinese Society of Clinical Oncology (CSCO) clinical practice guidelines for common malignant tumors**

| Evidence Characteristics | | | CSCO Expert Consensus |
| --- | --- | --- | --- |
| Category | Level | Source |  |
| 1A | High | Rigorous meta-analyses, large randomized controlled studies | Unanimous consensus (support ≥80%) |
| 1B | High | Rigorous meta-analyses, large randomized controlled studies | Basic consensus (support 60%–<80%) |
| 2A | Slightly Low | Meta-analyses of average quality, small randomized controlled studies, well-designed large retrospective studies, case-control studies | Unanimous consensus (support ≥80%) |
| 2B | Slightly Low | Meta-analyses of average quality, small randomized controlled studies, well-designed large retrospective studies, case-control studies | Basic consensus (support 60%–<80%) |
| 3 | Low | Single-arm clinical studies without control, case reports, expert opinions | No consensus, and highly controversial (support <60%) |
